# Role of Altered Metabolic Microenvironment in Osteolytic Metastasis

**DOI:** 10.3389/fcell.2020.00435

**Published:** 2020-06-05

**Authors:** Kerstin Tiedemann, Osama Hussein, Svetlana V. Komarova

**Affiliations:** ^1^Faculty of Dentistry, McGill University, Montréal, QC, Canada; ^2^Shriners Hospitals for Children – Canada, Montréal, QC, Canada; ^3^Department of Surgery, Mansoura University Cancer Center, Mansoura, Egypt

**Keywords:** bioenergetics, metabolism, osteoclast, bone microenvironment, cancer, osteolysis, metabolic sensors

## Abstract

Metastatic bone disease is generally incurable and leads to pathological fractures, pain, hypercalcemia, spinal cord compression and decreased mobility. The skeleton is the major site of bone metastases from solid cancers, including breast and prostate carcinoma. Bone metastasis is facilitated by activation of bone-resorbing osteoclasts, terminally differentiated multinucleated cells formed by fusion from monocytic precursors. Cancer cells are known to produce specific factors that stimulate osteoclast differentiation and function. Of interest, cancer cells are also known to alter their own bioenergetics increasing the use of glycolysis for their survival and function. Such change in energy utilization by cancer cells would result in altered levels of cell-permeable metabolites, including glucose, lactate, and pyruvate. Osteoclast resorption is energy-expensive, and we have previously demonstrated that during differentiation osteoclasts actively adapt to their bioenergetics microenvironment. We hypothesize that altered bioenergetics state of cancer cells will also modify the bioenergetics substrate availability for the tissue-resident bone cells, potentially creating a favorable milieu for pathological osteolysis. The goals of this review are to analyze how metastasizing cancer cells change the availability of energy substrates in bone microenvironment; and to assess how the altered bioenergetics may affect osteoclast differentiation and activity.

## Introduction

Bone is a preferred organ for metastasis from many tumors, including breast, prostate, and lung carcinomas (Hernandez et al., 2018). Establishment of metastatic bone lesions is facilitated by resident osteoclasts, cells that specialize in bone destruction. Molecular signatures that allow successful integration of cancer cells in the bone microenvironment have been extensively investigated (Olechnowicz and Edwards, 2014; Hiraga, 2019), however, none of the identified factors fully explains the success of tumors in thriving in the bone. In this mini-review, we will explore if tumor-mediated changes in bioenergetic environment may contribute to supporting osteoclast formation and function.

Cancer cells are different from their somatic counterparts in many factors, including their bioenergetics. Warburg effect, an increased use of anaerobic glycolysis by cancer cells, has re-gained much attention in the recent years (Lunt and Vander Heiden, 2011; Liberti and Locasale, 2016). The benefits of upregulating glycolysis for cancer cells are not fully understood, since oxidation of one molecule of glucose into pyruvate and 36 molecules of ATP per glucose are produced lactate during glycolysis generates 2 molecules of ATP, while 36 molecules of ATP per glucose are produced during oxidative phosphorylation. However, glycolysis is also important for biosynthesis of nucleotides, lipids and amino acids, all required for cellular proliferation (Lunt and Vander Heiden, 2011). Many metabolites involved in glycolysis and Krebs cycle are transported by the solute-carrier gene (SLC) family of membrane-bound transporters (Markovich and Murer, 2004). Glucose transporters that belong to 2A family of SLCs, represent a rate-limiting step in glycolysis and are known to be strongly dysregulated in cancer cells (Adekola et al., 2012). Lactate and pyruvate are transported by monocarboxylate transporters MCT1-4 that belong to the 16A family of SLCs, and MCT1 and MCT4 are upregulated in several cancers (Jones and Morris, 2016; Li et al., 2018). Importantly, intracellular and extracellular pools of lactate and pyruvate interchange relatively fast (Quek et al., 2016), therefore changes in intracellular metabolite levels lead to corresponding changes in the extracellular environment of cancer cells.

All cells adapt their energy metabolism to changing levels of energy demands, as well as availability of energy substrates. AMP-activated protein kinase (AMPK) is stimulated by an increase in AMP/ATP ratio due to cells inability to meet the current energy demand (Finley and Haigis, 2009). AMPK acts to decrease metabolic expenditure and increase energy production (Gwinn et al., 2008). Mammalian target of rapamycin (mTOR) generally acts downstream of AMPK. Two mTOR complexes, mTORC1 (with raptor and PRAS40) and mTORC2 (with rictor, mSIN1, and proctor) have distinct roles. While mTORC1 regulates protein synthesis (Foster and Toschi, 2009) and the SLC-mediated metabolite transport (Taylor, 2014), mTORC2 is linked to cytoskeletal dynamics and cell survival (Gaubitz et al., 2015). The metabolic sensors, AMPK and mTOR are critical players in cellular adaptation to a varying bioenergetics environment.

The goal of this review is to examine how changes in extracellular glycolytic metabolites due to the presence of actively proliferating cancer cells may alter osteoclast metabolic support, differentiation and function.

## Bioenergetics Requirements of Osteoclasts

**FIGURE 1:**
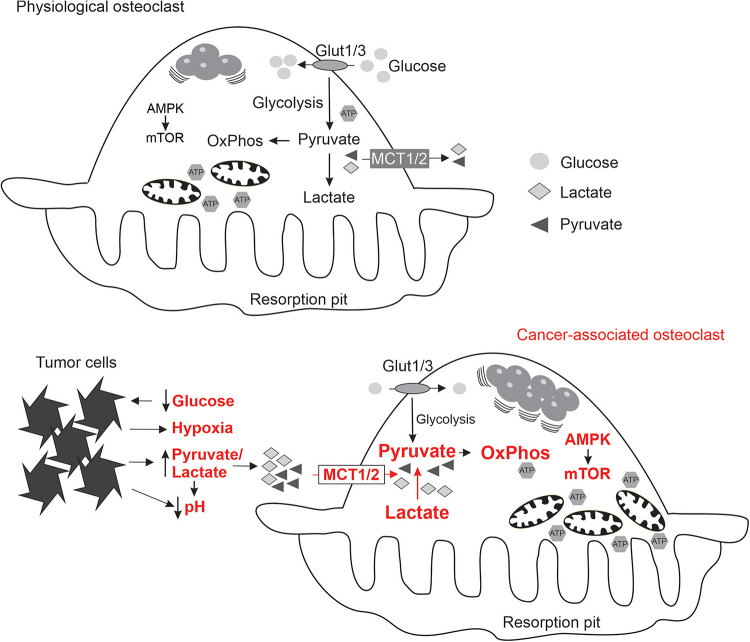
Schematics summarizing the state of energy metabolism in osteoclasts formed under physiological conditions (top), and the adaptive changes in osteoclasts (indicated in red) exposed to the local microenvironment modified by metastasizing cancer cells (bottom).

To understand how osteoclasts can be affected by the metabolic substrates, we need to consider the normal bioenergetic requirements of these cells at different stages of their differentiation and function. Osteoclasts are multinucleated cells formed by fusion of monocytes. Mature osteoclasts attach to bone matrix, forming a sealing zone, where proton pumps lower the extracellular pH to dissolve hydroxyapatite, and proteolytic enzymes are secreted to digest the organic matrix (Stenbeck, 2002). Osteoclasts survive for ∼7–10 days, after which they die primarily by apoptosis (Akchurin et al., 2008; Kopesky et al., 2014). Osteoclast differentiation and function place significant and varied demands for energy required for migration of monocytes for cell fusion, phospholipid synthesis for cell membrane growth, protein synthesis to gain resorptive capacity, action of ion pumps and secretion of proteolytic enzymes. To provide this energy, monocytes increase glucose and oxygen consumption within 24–48 h of exposure to RANKL (Kim et al., 2007), up-regulate metabolic enzymes involved in energy production (Czupalla et al., 2005), and generate abundant large mitochondria (Dudley and Spiro, 1961; Lemma et al., 2016; Figure 1). Mitochondrial biogenesis stimulated by peroxisome proliferator–activated receptor-c coactivator 1β (PGC-1β) is a pre-requisite of successful osteoclastogenesis (Ishii et al., 2009; Wei et al., 2010; Zeng et al., 2015; Zhang et al., 2018). During resorption, osteoclast glucose transport increases 2-fold (Williams et al., 1997) and mitochondria locate near resorption surface (Kawahara et al., 2009). ATP levels markedly increase during osteoclastogenesis (Le Nihouannen et al., 2010). AMPK and mTOR are important for osteoclast differentiation and function. Osteoclastogenesis is associated with changes in AMPK isoform composition (Fong et al., 2013) and AMPK negatively regulates early stages of osteoclast differentiation (Lee et al., 2010; Shah et al., 2010; Kang et al., 2013). Signaling through mTOR is critical for osteoclast formation and survival (Glantschnig et al., 2003; Sugatani and Hruska, 2005; Hu et al., 2016; Dai et al., 2017), while osteoclast fusion and cytoplasmic growth depend on mTOR-mediated Akt signaling (Tiedemann et al., 2017). Importantly, nutrient availability during osteoclast differentiation was shown to significantly affect AMPK, mTORC1 and mTORC2 complexes (Fong et al., 2013; Tiedemann et al., 2017). Thus, it is conceivable that changes in metabolic substrate accessibility due to the presence of proliferating cancer cells may directly affect osteoclast differentiation and function.

## Potential Effects of Alterations in Metabolic Environment on Osteoclasts

### Glucose

Glucose, transported by glucose transporters 1 and 3 (Kim et al., 2007), is the most effective bioenergetics substrate for supporting bone resorption (Williams et al., 1997). In the absence of glucose, fatty acids, ketone bodies, and lactate can support bone resorption at 20–30% of the levels achievable with glucose (Williams et al., 1997). Nevertheless, the dose-dependence of glucose effects is complex. An increase from less than 1 mM to 5–10 mM glucose was demonstrated to stimulate osteoclastogenesis (Kim et al., 2007), resorption (Williams et al., 1997), and osteoclastogenic signaling through p38 mitogen-activated protein kinase (Larsen et al., 2002) and calcium/calmodulin-dependent kinase II (CaMK II) (Larsen et al., 2005). In a mouse model of type 2 diabetes, moderate hyperglycemia [∼10 mM circulating glucose (Fernandez et al., 2001)] was associated with increased osteoclastogenesis (Kawashima et al., 2009). In contrast, high glucose concentrations inhibit osteoclastogenesis (Kim et al., 2007; Wittrant et al., 2008), which could be explained by metabolic effects, such as decreased oxygen consumption at higher glucose level [similar to the Crabtree effect observed in yeasts (Pfeiffer and Morley, 2014)], as well as osmotic effects (Botolin and McCabe, 2006). In the environment of highly glycolytic cancer cells, the ambient glucose levels would likely decrease, reducing its availability for osteoclastogenesis. Thus, decrease in glucose is unlikely to contribute to osteoclastogenic effects of cancer cells.

### Pyruvate

Several studies have investigated how pyruvate affects osteoclast formation. Addition of small amounts of pyruvate to media containing normal levels of glucose significantly increased osteoclastogenesis (Kim et al., 2007; Fong et al., 2013), resulting in formation of large osteoclasts that contained more nuclei per cell (Fong et al., 2013; Tiedemann et al., 2017). Of interest, only when added in relatively small amounts, between 1 and 2 mM (Fong et al., 2013; Tiedemann et al., 2017) and 5 mM (Kim et al., 2007), pyruvate was effective in promoting osteoclast formation. Addition of low pyruvate concentrations stimulated osteoclast mitochondrial activity, leading to a metabolic shift toward oxidative phosphorylation, and an increase in cellular [ATP] (Kim et al., 2007; Fong et al., 2013). Pyruvate caused an inhibition of AMPK and an activation of mTOR/raptor complex leading to facilitated protein synthesis and cytoplasmic growth (Fong et al., 2013; Tiedemann et al., 2017). MCT1, 2, and 4 for lactate and pyruvate are expressed by osteoclasts (Imai et al., 2019). MCT2 has the highest affinity for both pyruvate (Km ∼0.1 mM) and lactate (Km ∼0.7 mM), compared to MCT1 that has a Km value in millimolar range, and MCT4, affinity of which is even lower (Halestrap, 2012). Low concentration of MCT inhibitor or deletion of MCT1 were shown to potentiate osteoclastogenesis, while high concentration of MCT inhibitor or deletion of MCT2 prevented osteoclast formation (Imai et al., 2019). Another important issue with the interpretation of pyruvate effects was highlighted by Long and Halliwell (2009), who demonstrated that addition of pyruvate dramatically affects the media levels of hydrogen peroxide, which in turn affects osteoclastogenesis (Le Nihouannen et al., 2010). Nevertheless, no anti-oxidative effects were observed after addition of small amounts of pyruvate (Fong et al., 2013). Increase in glycolysis due to Warburg effect in cancer cells can lead to increased production of pyruvate that can in turn be transported to the extracellular space (Doherty and Cleveland, 2013; Quek et al., 2016), and provide increased bioenergetic support for osteoclast formation.

### Krebs Cycle Metabolites

Krebs cycle occurs in the mitochondria, however, several of its metabolites, including citrate, succinate, malate, oxaloacetate, fumarate, and α-ketoglutarate can be transported through the cell membrane by sodium-dependent SLC13 transporters (Markovich and Murer, 2004; Pajor, 2014). Citrate in particular gained a lot of interest, since its extracellular levels vary in diseases (Huang et al., 2020). Of particular interest is reported reduction in plasma citrate levels in prostate and lung cancers that readily metastasize to bone (Rocha et al., 2011; Dittrich et al., 2012), as well as in osteoporosis, in which citrate is also reduced in bone (major citrate reservoir) (Chen et al., 2018). Extracellular citrate affects osteoclastogenesis, however, contradictory outcomes were reported. Similar to pyruvate, 1–2 mM of sodium citrate was shown to enhance osteoclastogenesis (Fong et al., 2013). However, potassium citrate dose-dependently inhibited osteoclast formation at similar concentrations (Granchi et al., 2017). Importantly, osteoclast inhibition was also observed upon addition of potassium ion K^+^ (KCl) (Yeon et al., 2015), suggesting that the effect of citrate may depend on media composition. Another potentially important link to Krebs cycle metabolites was proposed through glutamate metabolism. The glutamine transporter from SLC family 1a5 and glutaminase-1 converting glutamine to glutamate were shown to increase during osteoclastogenesis, leading authors to speculate that glutamate can be converted to α-ketoglutarate, which fuels energy metabolism (Indo et al., 2013). However, actively secretion of glutamate by osteoclasts was also demonstrated (Morimoto et al., 2006; Seidlitz et al., 2010). Thus, while glutamate likely plays an important role during osteoclastogenesis, it is difficult to conclude if its main action is relevant to energy metabolism. No information about other Krebs cycle intermediary is currently available. Thus, while the decreased citrate levels associated with cancer may affect osteoclastogenesis, the outcome of these interactions is uncertain and likely influenced by the localized cell microenvironment.

### Mitochondria

The presence of highly proliferative cancer cells results in hypoxic microenvironment (Al Tameemi et al., 2019), which stimulates osteoclast differentiation and supports resorption (Arnett, 2010; Knowles, 2015). Hypoxic environment leads to a surprising improvement of mitochondrial function and ATP production in osteoclasts (Knowles, 2015), which may be due to reduction in proton leak and uncoupled respiration noted in mitochondria exposed to low oxygen tension (Gnaiger et al., 2000). Mitochondria activity is also linked to the production of reactive oxygen species (ROS) such as peroxide and superoxide (Knowles, 2015). ROS generate oxidative stress, which is counteracted by cellular glutathione (GSH) producing its oxidized form, glutathione disulfide (GSSG). Oxidative stress has a bimodal effect on osteoclasts: while moderate stress resulting in GSH/GSSG decrease is stimulatory for osteoclastogenesis, severe stress leading to glutathione depletion inhibits resorption and limits osteoclast lifespan (Kim et al., 2006; Le Nihouannen et al., 2010; Domazetovic et al., 2017). Cancer cells also actively modulate their oxidative microenvironment by secreting antioxidants, such as peroxiredoxin 4 (Rafiei et al., 2015; Tiedemann et al., 2019), suggesting tumor-associated oxidative stress may differ for tumor types and stages of their growth. Additionally, oxidative stress is also induced by chemotherapy, such as doxorubicin (Rana et al., 2013). Thus, hypoxia and potentially oxidative stress generated by cancer cells may provide a microenvironment that supports osteoclastogenesis.

### pH and Lactate

Changes in pH are integral to the metabolic glucose processing. Anaerobic glycolysis results in acidification due to production of two molecules of lactic acid per each glucose (lactic acidosis), while complete mitochondrial oxidation of glucose generates six protons per glucose. Active metabolism of proliferating cancer cells is well recognized to produce acidic extracellular environment (Corbet and Feron, 2017). Acidification is also known to be a prerequisite of successful osteoclastogenesis (Arnett, 2010; Yuan et al., 2016; Arnett and Orriss, 2018). Osteoclasts sense extracellular acidosis through the G-protein coupled receptors, including ovarian cancer G-protein-coupled receptor 1 (OGR1) (Yang et al., 2006; Pereverzev et al., 2008; Li et al., 2009; Yuan et al., 2014) and T cell death-associated gene 8 (TDAG8) (Hikiji et al., 2014). In addition, osteoclasts express acid-sensitive ion channels (ASIC) (Jahr et al., 2005; Li et al., 2013). Acidosis was demonstrated to induce nuclear translocation of key osteoclastogenic transcription factor, nuclear factor of activated T cells 1c (NFATc1) (Komarova et al., 2005; Li et al., 2013) resulting in improved osteoclast formation (Granchi et al., 2017), resorptive activity (Komarova et al., 2005; Ahn et al., 2016), and survival (Pereverzev et al., 2008). Lactate was shown to be taken up by osteoclast precursors via MCT1 and to drive oxidative phosphorylation thereby facilitating bone resorption (Lemma et al., 2017). Thus, tumor-associated tissue acidosis and increased extracellular lactate can be expected to promote osteoclast differentiation and activity.

### Metabolic Adaptation of Osteoclasts to Cancer Microenvironment

Metastasizing cancer cells generate unique bioenergetics microenvironment: while normal substrates, glucose and oxygen, are consumed by cancer cells, and therefore not available for osteoclasts, cancer cells generate alternative substrates such as pyruvate and lactate. In addition, acidic, hypoxic and potentially oxidative environment is uniquely supportive for osteoclastogenesis. To successfully perform in this altered microenvironment, osteoclasts need metabolic sensors to adapt their energy metabolism (Figure 1). We have shown that soluble factors produced by breast cancer cells induce a change in osteoclast mTOR signaling (Hussein et al., 2012). Moreover, targeting mTOR with rapamycin in the mouse model of experimental bone metastases resulted in a significant attenuation of cancer-induced osteolysis (Hussein et al., 2012; Abdelaziz et al., 2014), but had minimal effect on osteoclasts in the cancer-free bones of the same animals (Abdelaziz et al., 2015). These findings suggest that metabolic sensors are central for osteoclast adaptation to the metastatic microenvironment, and may represent therapeutic targets reviewed in the following section.

## Effect of Bioenergetics Targeting Therapies on Bone Metastasis

Therapeutics targeting metabolic sensors, such as metformin for AMPK and rapamycin for mTOR, have been successfully used for many years in a number of conditions including diabetes (Kezic et al., 2018) and organ transplantation (Augustine et al., 2007; Nguyen et al., 2019). In this section we attempted to review available evidence for the effectiveness of metformin and rapamycin and their analogs in preventing and/or controlling bone metastases.

### Metformin

Metformin is an anti-diabetic drug that activates AMPK (Faubert et al., 2015). In cancer cells, loss of AMPK induced a typical Warburg effect in transformed and non-transformed cells (Faubert et al., 2013), and promoted unchecked mTORC1 activity (Inoki et al., 2003). Activation of AMPK has multiple anti-tumor effects (Schulten, 2018), particularly in colorectal and prostate cancer patients (Coyle et al., 2016). In bone, in addition to its role in osteoclastogenesis, AMPK reduced the expression of osteoclastogenic cytokine RANKL (Lee et al., 2010; Wang et al., 2013; Cuyàs et al., 2017). While reports of treatment of bone metastases with metformin are sparse (Wang et al., 2013), a reduction in growth of primary tumor and metastases was demonstrated in a model of castration-resistant prostatic carcinoma upon treatment with metformin and simvastatin (Babcook et al., 2014). Limited number of reports regarding the effectiveness of metformin can be explained by the study that demonstrated that metformin looses its ability to activate AMPK in hypoxic conditions, which are commonly associated with growing tumor (Garofalo et al., 2013).

### Rapamycin and Its Analogs

In preclinical models of breast cancer bone metastases, rapamycin reduced osteolysis and bone pain, and improved animal survival (Hussein et al., 2012; Abdelaziz et al., 2014). Everolimus, a rapamycin analog more selective toward mTORC1 pathway, was also effective in preventing or treating experimental bone metastases from breast (Simone et al., 2015; Browne et al., 2017), prostate (Morgan et al., 2008), and lung (Yu et al., 2014) cancers. Several clinical trials evaluated the effectiveness of everolimus therapy in the treatment of hormone-receptor positive, Her2/Neu negative advanced breast cancer patients. A phase III, double-blind, randomized international BOLERO-2 trial compared the combination of anti-estrogen aromatase inhibitor exemestane with everolimus or placebo in postmenopausal women with advanced breast cancer. In addition to increasing progression-free survival (Yardley et al., 2013), everolimus markedly decreased levels of bone resorption biomarkers in patients with or without bone metastases (Gnant et al., 2013). RADAR clinical trial reported the effectiveness of everolimus in increasing the time to progression in a phase II double-blind, placebo-controlled, randomized discontinuation study in advanced breast cancer patients with bone metastases only (Maass et al., 2013). Thus, targeting mTOR appears promising in preclinical and clinical studies.

## Overall Conclusion

The presence of cancer cells in the bone microenvironment likely results in local hypoglycemia and hypoxia. However, an increased glycolysis due to the Warburg effect in cancer cells may provide alternative metabolic substrates such as superfluous pyruvate and lactate. Adaptation of osteoclasts to such environment likely require the activity of metabolic sensors AMPK and mTOR. Importantly, osteoclasts are known to successfully adapt their mitochondrial function to conditions of hypoxia, which in osteoclasts stimulates ATP production, differentiation and function (Knowles, 2015). Acidification is another cancer-driven change in the microenvironment that is known to be specifically stimulatory for osteoclast formation and function (Arnett and Orriss, 2018). Thus, osteoclasts formed in the osteolytic tumor lesions are likely different from physiologically formed in their reliance on alternative metabolic substrates, adjusted activity of metabolic sensors, and unusual mitochondria function. Of interest, the combination of syrosingopine-mediated inhibition of MCT1 and 2 with metformin was recently demonstrated to result in synthetic lethality for cancer cells (Benjamin et al., 2018). We suggest that such drug combinations may target both cancer cells and cancer-supportive osteoclasts alleviating destructive and painful bone metastasis.

## Author Contributions

KT and SK conceived the study, researched, and summarized the preclinical studies. OH researched and summarized the clinical studies. All authors contributed to manuscript writing and approved the final version of the manuscript.

## Conflict of Interest

The authors declare that the research was conducted in the absence of any commercial or financial relationships that could be construed as a potential conflict of interest.

## Abbreviations

AMPK: AMP-activated protein kinase
mTOR: Mammalian target of rapamycin
MCT: Monocarboxylate transporters
PGC-1 β: Peroxisome proliferator–activated receptor-c coactivator 1 β
RANKL: Receptor activator of nuclear factor kappa B-ligand
SLC: Solute carrier transporters.
